# Ultra-Marathon Athletes at Risk for the Female Athlete Triad

**DOI:** 10.1186/s40798-015-0027-7

**Published:** 2015-09-09

**Authors:** Lindy-Lee Folscher, Catharina C Grant, Lizelle Fletcher, Dina Christina Janse van Rensberg

**Affiliations:** 1 Section Sports Medicine, Faculty of Health Sciences, University of Pretoria, Hatfield, Pretoria, 0002 South Africa; 2 Department of Statistics, University of Pretoria, Hatfield, Pretoria, 0002 South Africa

**Keywords:** Female athlete triad, Ultra-marathon runners, Risk of female athlete triad, Knowledge of female athlete triad

## Abstract

**Background:**

Worldwide female participation in ultra-endurance events may place them at risk for the female athlete triad (FAT). The study objectives were to establish triad knowledge, occurrence of disordered eating and triad risk amongst participants of the 2014 89-km Comrades Marathon event.

**Methods:**

A survey utilising the Low Energy Availability in Females questionnaire (LEAF-Q) and Female Athlete Screening Tool (FAST) questionnaire was conducted on female participants in order to determine the risk. In addition, seven questions pertaining to the triad were asked in order to determine the athlete’s knowledge of the triad. Athletes were requested to complete the anonymous questionnaire after written informed consent was obtained while waiting in the event registration queues. Statistical analyses included Pearson product–moment correlations, chi-square tests and cross-tabulations to evaluate associations of interest.

**Results:**

Knowledge of the triad was poor with 92.5 % of participants having not heard of the triad before and most of those who had, gained their knowledge from school or university. Only three athletes were able to name all 3 components of the triad. Amenorrhoea was the most commonly recalled component while five participants were able to name the component of low bone mineral density. Of the 306 athletes included in the study, 44.1 % were found to be at risk for the female athlete triad. One-third of participants demonstrated disordered eating behaviours with nearly half reporting restrictive eating behaviours. There is a significant association between athletes at risk for the triad according to the LEAF-Q and those with disordered eating (*χ*^2^(1) = 8.411, *p* = 0.014) but no association (or interaction) between triad knowledge and category (at risk/not at risk) of LEAF-Q score (*χ*^2^(1) = 0.004, *p* = 0.949). More athletes in the groups with clinical and sub-clinical eating disorders are at risk for the triad than expected under the null hypothesis for no association.

**Conclusions:**

Only 7.5 % of the female Comrades Marathon runners knew about the triad despite 44.1 % being at a high risk for the triad. Therefore, education and regular screening programmes targeting these athletes are overdue. Postmenopausal athletes are at particularly high risk for large losses in bone mass if they experience chronic energy deficiency and hence require special focus.

## Key points

- Knowledge of the triad is very poor amongst South African ultra-marathon runners.
- There are many misconceptions about menstrual function and exercise training amongst these endurance athletes.
- High rates (32 %) of disordered eating exist amongst female Comrades Marathon runners.
- 44.1 % of Comrades Marathon runners are at risk for the triad.

## Background

Female sports participation and ultra-marathon running has increased exponentially worldwide over the past three decades [1, 2]. This is reflected by female finishers of the annual 89-km Comrades Marathon run between Pietermaritzburg and Durban, South Africa which increased from 33 in 1980 to 2429 in 2014 [3]. Sport participation benefits weight management, chronic disease, high risk behaviour reduction, improved mental health, enhanced self-esteem and academic performance [4]. Unfortunately, intense training poses risks for female endurance athletes [5].

The female athlete triad (FAT) is a syndrome involving an interplay between low energy availability, menstrual dysfunction and altered bone mineral density [6]. Endurance runners are at increased risk posed by high energy demands of their training regimen and a potential desire for leanness for improved performance [7, 8]. Studies of collegiate and young adult endurance runners demonstrated higher prevalence of low bone mineral density (39.8 vs 10 %) amongst elite and recreational runners compared to non-runners [9].

Adequate dietary intake supplies energy for numerous physiological processes. This requires excess energy after exercise energy expenditure. If excessive exercise or dietary restriction reduces available energy below 30 kcal.kg^–1^ of fat free mass per day, physiological processes are compromised with suppression of reproductive function and new bone formation and consequent infertility, osteoporosis and stress fractures [5, 6, 10]. Adequate nutrients are also essential to maintain muscle mass, repair exercise-induced tissue damage and recover from illness and injury. Hence, energy deficiency results in injuries and poor recovery from intense training [10].

Low energy availability from excessive exercise expenditure, poor nutritional habits or eating disorders is the central pathological process of the triad [8, 11]. The Female Athlete Triad Screening Tool (FAST) was designed and validated to identify females at risk for disordered eating. The validated Low Energy Availability in Females questionnaire (LEAF-Q) determines triad risk by assessing injury history and gastrointestinal and menstrual function [12, 13].

Previous prevalence studies have investigated adolescents and collegiate cross country and recreational distance runners (5–20 km), demonstrating rates of at least one triad component between 16 and 78 % [14, 15]. Prevalence data amongst ultra-marathon athletes is limited. Study results from the Two Oceans Ultra-marathon athletes found disordered eating in 14.7 % of those interviewed [16]. The self-loathing subscale used utilised only four questions and more comprehensive screening may have uncovered higher rates. Additional prevalence data is also required amongst recreational athletes [15].

Treatment of the triad is complex due to psychological and performance-related aspects therefore prevention should be prioritised and requires education of athletes and coaches, which is often sorely lacking. An Australian study demonstrated that only 10 % of 18–40-year-old exercisers interviewed were able to name all triad components [8]. No studies were found on triad knowledge amongst South African female ultra-marathon runners.

The Comrades Marathon, considered “the holy grail” of road running amongst South Africans, requires consistent weekly training of 80–200 km during the 6 months preceding the event, which places these athletes at risk for low energy availability and its health consequences. The primary objective of this study was to establish knowledge of the triad amongst these athletes and then determine their level of risk.

## Methods

A cross-sectional, descriptive study utilising a questionnaire survey was conducted after ethical approval (159\2014) from the Faculty of Health Sciences, University of Pretoria, Ethics Committee. All study procedures followed were in accordance with the Helsinki Declaration of 1975, as revised in 2013. Registered female participants for the 2014 Comrades Marathon were invited to be study participants during race registration at the International Conference Centre, Durban, KwaZulu-Natal, South Africa. Participants provided written informed consent prior to questionnaire completion and no participation incentives were offered.

### Data Collection

Females waiting in the registration queue were arbitrarily approached and asked to complete an anonymous questionnaire after reading the information and consent form. Three-hundred and seventy-one athletes were approached and 351 agreed to participate. Forty-five incomplete questionnaires were excluded from analysis. Three-hundred and six completed questionnaires, of which 42 were from international runners, were included in the sample representing 12.6 % of the 2429 females who completed the 2014 event. Research assistants were available to clarify any questions while participants completed the paper-based questionnaires. On completion, questionnaires were collected and placed in a sealed box. Questionnaires were marked and scored by the principle investigator according to the scoring tool for the LEAF-Q and FAST questionnaires.

### Questionnaire Data

Questionnaire data included age, self-reported weight and height, training mileage, average training pace, number of Comrades Marathons completed and fastest finishing time. Participants were asked about their familiarity with the FAT, and if so, the source of their knowledge. In order to identify athletes at risk for the triad and disordered eating behaviours, the LEAF-Q and FAST were incorporated into the questionnaire. The FAST, developed to identify disordered eating behaviours in athletes, is reliable with a high internal consistency (Cronbach’s *α* = 0.87). Higher FAST scores are associated with eating disorders as identified by the Eating Disorder Examination Questionnaire (0.60, *p* < 0.05) and Eating Disorder Inventory (0.89, *p* < 0.001) [13]. The 25 item LEAF-Q was developed to identify athletes at risk for low energy availability by utilising subsets of gastrointestinal symptoms, injury frequency and menstrual dysfunction. The LEAF-Q was validated in endurance athletes (Cronbach’s *α* ≥ 0.71) with scores ≥8 producing a sensitivity of 78 % and specificity of 90 % to correctly classify energy availability and/or reproductive function and/or bone health [12].

### Statistical Analysis

Data from completed questionnaires were analysed using IBM Statistics SPSS 22. Descriptive statistics were calculated and Pearson product–moment correlations and chi-square tests were used to assess associations of interest.

## Results

### Participant Characteristics

Comrades Marathon 2014 participants are older athletes with a range of ultra-marathon experience, performance and training load as shown in Table 1.

**Table 1:** Demographic data, training load, running experience and performance

	Mean	Standard deviation	Median	[Min; max]
Age (years)	39.45	7.97	40.00	[21;65]
Weight (kg)	59.15	6.97	59.00	[42;83]
Height (m)	1.65	0.08	1.65	[1.3;1.85]
Number of Comrades Marathons completed	2.24	3.03	1.00	[0;22]
Fastest Comrades Marathon (89 km) time (hr:min)	10:05	1:34	10:35	[7:02;12:00]
Average training during peak training (h/week)	8.66	3.14	8.00	[1.5;20]
Average training pace (min/km)	5.55	0.70	6.00	[4.20;9.00]
Fastest standard marathon (42 km) time in last 6 months (hr:min)	4:06	0:64	4:19	[2:42;5:59]

### Demographic Data

#### Knowledge of the Female Athlete Triad

Most participants had never heard of the triad before (Table 2) and most of those who had, gained their knowledge from school or university. Only three athletes were able to name all three components of the triad. Amenorrhoea was the most commonly recalled component while five participants were able to name the component of low bone mineral density.

**Table 2:** Knowledge of the female athlete triad amongst female participants in the 2014 Comrades Marathon

Question	Frequency	Percentage (%)
1. Familiarity with triad	23	7.5
2. No previous knowledge	283	92.5
3. If yes, where did you hear about it?		
Sports magazine	2	0.65
Running book	2	0.65
University/school	13	4.2
Internet	3	1.0
Coach	1	0.35
Race expo	2	0.65
Not applicable	283	92.5
4. Ability to name components		
Unable to name any	286	93.5
Of the 20 participants attempting to name components:		
Able to name only one	6	2.0
Able to name two	7	2.2
Able to name all three	3	1.0
Incorrect	4	1.3

#### Knowledge of the Health Implications for the Female Athlete Triad

Of the 23 participants who had heard of the triad before only 12 could name 2 negative health consequences. Seven could correctly name 1 and 4 were unable to name any. The most commonly mentioned negative consequence was osteoporosis.

#### Self-Assessed Risk for the Triad

Only 2.3 % of participants thought they were at risk for the triad. The overwhelming majority (83.3 %) were uncertain whether they were at risk and 14.4 % believed that they were not.

#### Beliefs About Female Menstrual Function with Training

There was an even split (48.4 %, respectively) between participants who believed it normal to cease menstruation with heavy training and those who did not, while the remainder (3.2 %) expressed uncertainty. Even though many participants stated that amenorrhoea was a normal consequence of heavy training, the majority (83.3 %) believed it to be unhealthy.

### Key Components of the LEAF Questionnaire

Table 3 highlights some key LEAF-Q responses. Many athletes reported injuries, the most common being muscular strain/tears and iliotibial band friction syndrome. Menstrual changes were common with increased exercise with one-fifth of those capable of menstruation reporting amenorrhoea with heavy training loads. Gastrointestinal disturbances were less frequent than menstrual disturbances and injuries.

**Table 3:** Responses to key components of LEAF-Q as reported by female participants of the 2014 Comrades Marathon

LEAF questionnaire component	Frequency	Percent
1. Injury history		
Number of days lost from participation due to injury in past year:		
0	120	39.2
1–7	91	29.7
8–14	39	12.8
15–21	24	7.8
≥22	32	10.5
Most common injuries reported:		
(Respondents could choose more than one)		
Muscular strain/tear	38	11.8
Iliotibial band friction syndrome (ITB)	36	11.2
Knee injury	24	7.4
Hamstring injury	18	5.6
Achilles tendonitis/ankle injury	14	4.3
Plantar fasciitis/foot injury	14	4.3
Stress fractures	11	3.4
2. Menstrual function		
Exercise-related menstrual changes:		
Bleed less	79	72.5
Menstruation stops	22	20.2
Increased bleeding	8	7.3
Number of periods in the last year (if still menstruating)		
≥9	184	60.2
5–8	20	6.6
0–2	2	0.7
Secondary amenorrhoea	71	23.2
Current and not menopausal		
Postmenopausal	14	4.6
Hysterectomy	30	9.8
3. Gastrointestinal disturbances		
Abdominal bloated/gaseous when not having periods:		
Daily–weekly	55	14.7
Seldom	76	24.8
Rarely or never	185	60.5
Cramps/stomach ache not related to your menstruation:		
Daily–weekly	42	13.8
Seldom	57	18.6
Rarely or never	207	67.6

### LEAF-Q Scores

Nearly half (44.1 %) of participants are classified as being at risk for the triad according to their LEAF-Q scores.

### FAST Questionnaire Scores

Table 4 shows that according to FAST questionnaire responses, almost one-third of participants are at risk for low energy availability related to their eating behaviours. The majority of participants believed their running performance was related to their weight with 60.5 % expecting performance improvements with weight reduction and 67.4 % worrying that weight gain would impair their performance. Restrictive eating was reported by nearly half of participants with 47.7 % controlling fat and calorie intake and 44.5 % limiting carbohydrate intake. Many participants reported using exercise for weight management with 50.3 % reporting that they trained intensely in order to avoid weight gain and 71.8 % stating that they would worry about weight gain if unable to exercise or injured and therefore restrict their calorie intake. A large percentage (68.3 %) considered sport participation as important for their self-esteem.

**Table 4:** Number of female athletes with disordered eating according to FAST questionnaire results

	Frequency	Percent	Mean FAST score	SD
No eating disorder	208	68.0	61.79	9.59
Subclinical eating disorder	82	26.8	82.70	4.68
Clinical eating disorder	16	5.2	100.13	6.04

### Correlative Analysis

Table 5 shows that higher LEAF-Q scores correlate negatively with faster race performance, while higher FAST scores correlate positively with LEAF-Q scores, weight and BMI.

**Table 5:** Summary of bivariate correlations

Variables	Pearson coefficient	*p* for *H*_0_: *ρ* = 0
LEAF-Q score and Comrades performance	−0.159	0.029
FAST score and LEAF-Q score	0.244	<0.001
FAST score and weight	0.198	0.001
FAST score and BMI	0.186	0.001

### Cross-tabulation

The cross-tabulation of FAST and LEAF-Q categories (Table 6) demonstrates more athletes at risk for the triad in the groups with clinical and sub-clinical eating disorders than expected under the null hypothesis of no association (count vs. expected count).

**Table 6:** Cross-tabulation of FAST and LEAF-Q score categories

FAST score category:		Not at risk for triad according to LEAF-Q score	At risk for triad according to LEAF-Q score	Total
No eating disorder	Count	125	83	208
	Expected count	116.2	91.8	
	Column %	73.1 %	61.5 %	68 % of 306
Subclinical eating disorder	Count	42	40	82
	Expected count	45.8	36.2	
	Column %	24.6 %	29.6 %	26.8 % of 306
Clinical eating disorder	Count	4	12	16
	Expected count	8.9	7.1	
	Column %	2.3 %	8.9 %	5.2 % of 306

Pearson chi-square = 8.411; degrees of freedom = 2; *p* = 0.015

Cross-tabulation of triad knowledge (Yes/No) by LEAF-Q score (Not at risk/At risk) demonstrated no association between triad knowledge and LEAF-Q score category (*χ*^2^(1) = 0.004, *p* = 0.949).

## Discussion

Ultra-endurance sport attracts older athletes due to age restrictions on participation and possibly the fact that many long distance athletes compete in shorter distances before attempting ultra-endurance events. The mean age of athletes studied was 39 years. Marathon finishing times of participants demonstrated that both elite runners and recreational athletes were included in the sample, increasing the likelihood of results being representative of the female ultra-marathon running community.

A disturbingly small percentage (7.5 %) of participants knew about the triad. The majority (80 %) of these were international runners from Europe, USA or Australia. The most knowledgeable athletes had obtained their knowledge at school or university. It is likely that these are younger athletes with recent education which has been influenced by the Female Athlete Triad Coalition and other regulatory bodies’ drive for pre-participation evaluation and education [11, 17]. This lack of knowledge is concerning since endurance athletes are at increased risk due to the high volume training associated with ultra-marathon preparation and possible inadvertent low energy availability [18]. Only 1 % of participants could name all three triad components which is lower than the 10 % reported by Australian women [8]. Knowledge of negative health implications of the triad was equally poor with only 3.9 and 2.3 % able to name one and two negative consequences, respectively. Similar rates were found in an American study in which more than 90 % of female high school track athletes were unable to link menstrual irregularity with negative bone consequences [17].

Very few participants believed they were at risk for the triad, possibly since 83.3 % were unsure what the triad was. The 44 participants who explicitly stated that they were not at risk demonstrate blissful ignorance and are therefore unlikely to pursue further understanding of the triad. It could be argued that simply engaging in endurance training places female athletes at risk, which may be mitigated by education on nutritional intake and careful dietary monitoring. However, female endurance athletes often seek to reduce body weight without seeking professional guidance, resulting in chronic energy deficiency [19].

The fact that 48.4 % of participants believed amenorrhoea to be normal with heavy training is concerning and higher than the 35 % reported by Australian women [8]. These athletes are unlikely to seek medical attention for amenorrhoea, resulting in a missed opportunity for early intervention. In addition, this belief may cause athletes to use amenorrhoea as an indicator of hard training, driving them towards this “marker” of adequate training. Amenorrhoea may also be considered “convenient” by athletes since they are relieved of the discomfort caused by menstruation. Conversely, low energy might initially result in subclinical menstrual disorders still placing athletes at risk since these disorders go undetected when athletes use amenorrhoea as a “red flag” for excessive training [20, 21].

Fifty percent of women capable of normal menstrual cycles reported changes with increasing exercise, similar to other studies that reported 43–78 % of athletes with menstrual dysfunction, exacerbated by increasing training [16, 21, 22]. Menstrual dysfunction is a useful triad screening tool but was not applicable to many older athletes due to Mirena® use, postmenopausal status or previous hysterectomy [11, 21, 23]. Postmenopausal females are already in a hypo-estrogenic state and at risk for osteoporosis. Energy deficits with resultant oestrogen deficiency leads to abnormal bone remodelling placing energy deficient postmenopausal athletes at risk [21]. Protective effects of weight-bearing exercise on postmenopausal bone loss may be lost with high training volumes and therefore more research may be required on these vulnerable athletes [24].

If triad knowledge is so poor amongst this group of athletes, they probably have inadequate nutritional knowledge to prevent inadvertent energy deficiency. Previous studies revealed that female endurance athletes often consume an incorrect macronutrient balance causing calorie deficiencies [25, 26]. One-third of athletes were categorised as having disordered eating by FAST scores—higher than the 14.7 % reported by Micklesfield et al. in Two Oceans athletes but similar to rates reported by Cobb et al. (25.5 %) amongst competitive distance runners [16, 23]. The majority of these athletes demonstrate a subclinical eating disorder with only 5.2 % meeting clinical eating disorder criteria, only marginally higher than general population rates (2–4 %) [27]. There were more athletes than expected with disordered eating behaviours in athletes at risk according to LEAF-Q scores confirming the interaction between disordered eating and triad risk. Since disordered eating is associated with low bone mineral density in competitive female distance runners without menstrual irregularity, screening of these athletes is advisable [23]. Dietary restraint is the disordered eating behaviour most closely associated with bone mineral loss and is therefore an important risk factor [28]. Nutritional trends amongst athletes have drastically changed in recent years following publicised low carbohydrate/high fat diets. Nearly 45 % of participants reported carbohydrate restriction. This is likely due to proposed benefits of improved body composition and performance without adequate input from a dietician thereby placing themselves at greater risk for calorie deficits. While low carbohydrate/high fat diets assist in weight loss, performance benefits for endurance athletes as well as the long-term health implications of such diets may be questioned. In addition to this, appetite often declines with intensive training and cannot be used as an indicator of energy requirements for endurance athletes [19]. Therefore, nutritional assessment and support may be beneficial for this group.

Weight control may be the motivation for or consequence of endurance exercise. Half of participants reported weight management as a motivation for training and 68 % expressed performance-related weight beliefs. Gibbs et al. demonstrated an association between a high drive for thinness amongst exercising women and energy deficiency, highlighting the need to screen athletes for energy and menstrual status if direct measurement is not feasible [29]. The high proportion demonstrating “weight consciousness” highlights the urgent need for screening of these athletes to detect compulsive attitudes towards exercise as well as eating psychopathology which is directly linked to the triad and stress fracture risk [22].

Self-reported injury rates were fairly high with 59.1 % of participants reporting injuries in the preceding year. This injury rate is similar to other studies which report injury rates as high as 79.3 %. Typical injuries occur in the lower extremities and increase with increased training distance, participation in longer distance events and training on hard surfaces (mainly concrete). These factors are all relevant to this group studied [30–32]. The majority of reported injuries were overuse injuries in keeping with other studies on athletes with high training loads [31, 33].

LEAF-Q scores classified 44.1 % of participants at risk for the triad with a correlation between faster race performance and higher triad risk. Faster race performance, higher training volume and intensity have a strong association with bone stress injury and triad risk [18].

With the above in mind, prevention is considered “the ultimate treatment strategy” [5, 17]. Screening and educational interventions are the most effective preventative strategy amongst endurance athletes [6, 7, 11]. Education should include healthcare professionals managing athlete injuries/illnesses, club coaches, family and friends of athletes [5]. Unfortunately, knowledge of the triad is also poor amongst health-care professionals with only 48 % of USA physicians, 43 % of physical therapists, 32 % of athletic trainers and 8 % of coaches able to name all three components [34]. It is unlikely that the situation is any different amongst healthcare professionals treating these participants therefore education about the triad should be extended to include these professionals. In addition, governing bodies should ensure effective implementation of educational programmes [5]. Athletics South Africa (ASA), the governing body of running in South Africa, should become activists for the education of female athletes. Pre-participation medical screening questionnaires are required by organisers before entry into certain events and cover mainly chronic illness and medical/injury history. It is recommended that triad screening questions be included in these questionnaires.

### Strengths of the Study

All female athletes in the registration queue during sampling were requested to participate in the study to avoid selection bias from research assistants singling out “thin” athletes. South African runners as well as 42 international runners were asked to participate in order to get representation of the global female running community. Questions were simple and easy to understand.

### Limitations

The study is limited to female participants in the 2014 South African Comrades Marathon. The data is self-reported and therefore dependent on the understanding of the questions and honesty of completion. The 45 incomplete questionnaires were excluded from analysis. It was assumed that these were incomplete due to time constraints. It may however be that some questions were too sensitive, especially those about eating behaviours. It is therefore possible that occurrence rates of participants with clinical or subclinical eating disorders are underreported.

## Conclusions

This study highlights a group of athletes at high risk for the triad and calls for urgent educational interventions and regular screening for this group of athletes. Pre-participation screening should become mandatory and should be performed by race organisers or athletic governing bodies [17, 35]. This study population includes some older athletes entering menopausal years who are at particularly high risk for large losses in bone mass if exposed to chronic energy deficiency due to endurance exercise. More research may be required on this group of athletes.

### What is Already Known:

- The female athlete triad consists of an interaction between low energy availability, hormonal dysfunction with resultant menstrual disturbances and altered bone mineral density
- While ultra-marathon running is protective against chronic illness, runners are at risk for the negative health consequences of female athlete triad
- Disordered eating places athletes at risk for the triad particularly if engaged in high volume/high intensity training
